# Society Meetings

**Published:** 1908-01

**Authors:** 


### SOCIETY MEETINGS.

The Medical Society of the County of Montgomery celebrated its one hundred and first anniversary at the Central Hotel in the city of Amsterdam, Wednesday evening, December 12, 1907, where a banquet was served to the members and guests of the society. Dr. Charles Stover served as toastmaster and a number of well appointed speeches were made by Dr. F. C. Curtis, President of the Medical Society of the State of New York, who spoke for that body; Dr. D. C. Moriarta, of Saratoga, whose theme was the fourth district branch; Dr. C. B. Mosher, of Johnstown, who responded to the Medical Society of the County of Fulton; Dr. W. G. Macdonald, who spoke for the Albany Medical College; Dr. Andrew McFarlane, who took the place of Dr. A. Vander Veer; Dr. G. G. Lempe, who responded for the Medical Society of the County of Albany; Dr. George W. Bates, of Schenectady, who made an impromptu speech; and Dr. Hicks who also spoke without a subject. Those present at the dinner were:

Charles Stover, Archibald Gilbert, J. R. Fairbanks, W. R. Pierce, William M. Dwyer, D. M. Taylor, Richard R. Canna, John Warner Kniskern, T. G. Hyland, F. A. Husted, E. J. Collier, E. F. Bronk, E. C. LaPorte, J. B. Conant, E. K. Macomber, A. V. H. Smyth, Horace M. Hicks, L. A. Frazier, R. G. Johnson,

E. E. Rulison, C. F. Timmerman, S. H. French, George H. Loadwick and William J. Kline, Amsterdam; William C. McCulloch, Nelson Everest, S. H. Rabuck, George Lenz, C. A. Sternberg. William Clark Wood, Charles E. Paunael, Charles M. Lefler, W. S. Carnsey, John Edwards, R. J. Palmer, J. J. Beard, R. L. Ellithorp and S. C. Clemans, Gloversville; M. Somers, C. B. Mosher, F. Beebe, J. K. Young, C. B. Walrad, E. A. Stapleton, J. D. Vedder, J. W. Joslin and D. V. Still, Johnstown; F. E. Simons, Canajoharie; A. MacFarlane, Arthur J. Bedell, J. W. Vander Veer. A. H. Traver, H. Judson Lipes, George Gustave Lempe, Willis Goss Macdonald, Arthur Wells Elting, F. C. Curtis and H. E. Lomax, Albany; E. T. Rulison, George W. Bates, W. Faust, Schenectady; C. L. Meyer, Stone Arabia; W. J. Peddie, O. Z. Bouton, Fultonville; A. B. Foster, J. W. White, Fonda; H. W. Coffin, Glens Falls; Free J. Resseguie, D. C. Moriarta, Saratoga; H. R. Biggar, Glen; Peter L. Suits, Tribes Hill; W. D. Garlock, Little Falls; W. H. De La Mater, Minaville; Douglass Ayres, C. E. Congdon, Fort Plain.

---

THE Buffalo Academy of Medicine held meetings during November and December as follows:

*Section on Surgery.*—Wednesday evening, November 6. Program: Report of case of foreign body removed from the bladder with operating cystoscope, David E. Wheeler; (*a*) facial-hypoglossal anastomosis, with illustrations on the cadaver, George F. Cott; (*b*) vesicular diseases, Nelson W. Wilson.

*Section on Medicine.*—Tuesday evening, November 12. Program: Care of infant during first week of life, Dewitt H. Sherman; recurrent empyema, J. H. Pryor.

*Section on Pathology.*—Tuesday evening, November 19. Program: The shape and relations of the stomach; remonstrations of specimens, Professor A. T. Kerr, Professor of Anatomy, Medical Dept., Cornell University, Ithaca, N. Y.

*Section on Obstetrics and Gynecology.*—Tuesday evening, November 26. Program: (*a*) impotence in the female, J. Henry Dowd; (*b*) legal status of the unborn infant, Hon. John Lord O'Brian.

*Section on Surgery.*—Tuesday evening, December 3. Program: (*a*) electrical anesthesia, James E. King; (*b*) local anesthesia in minor and major surgery, Lawrence Hendee; (*c*) temporary anesthesia, H. B. Huver. (*d*) anesthesia by laughing gas, laughing gas and oxygen, chloroform and oxygen and ether by rectum, Prescott L. LeBreton; (*e*) post-operative complications of anesthesia, Roswell Park.

*Section on Medicine.*—Tuesday evening, December 10. Program: Fundamental principles of Therapeutics, John H. Musser, Philadelphia.

*Section on Pathology.*—Tuesday evening, December 17. Program: The placental transmission of tuberculosis, lantern slides, Alfred S. Warthin, Ann Arbor, Mich.

---

THE Medical Union at its annual meeting held Wednesday evening, December 18, 1907, elected the following named officers for the ensuing year: president, Vertner Kenerson; vice-president, Edward J. Meyer; secretary and treasurer, F. B. Rasbach.

---

THE Dunkirk and Fredonia Medical Society held a semiannual meeting and banquet at the Erie Hotel, Dunkirk, December 11, 1907. Sixteen members were present. At the business meeting Dr. C. H. Richards read a paper. The following named officers were elected for the ensuing year: president, William J. Perish of Fredonia; vice president, John A. Weidman of Dunkirk; secretary and treasurer, Joseph Rieger of Dunkirk.

---

THE Medical Society of the County of Chautauqua, held its annual meeting at the Erie Hotel, Dunkirk, Tuesday, December 10, 1907.

The morning session was called to order at 11 o'clock by Dr. C. H. Richards of Dunkirk, president of the society. At this session three new members were taken into the society, namely, R. B. Blanchard of Jamestown, May Gibson Waters of Hamlet, W. C. Duke of Fredonia.

At the afternoon session papers were read by C. H. Richards of Dunkirk, Morris N. Bemus of Jamestown, Fred C. Rice of Ripley, A. F. Soch of Fredonia, and C. W. Southworth of Forestville. Dr. DeLancey Rochester of Buffalo, president of the eighth district branch, read an interesting paper on prognosis and treatment of chronic valvular disease of the left heart. The following named officers were elected for the ensuing year: year: president, B. S. Sweetland of Jamestown; first vice-president, Morris N. Bemus of Jamestown; second vice-president, Edgar Rood of Westfield; secretary and treasurer, H. A. Eastman of Jamestown reëlected.

THE Rochester Academy of Medicine recently held meetings as follows:

Friday evening, November 15. Program: A working formula for the treatment of general peritonitis, W. B. Jones; report of a case of primary sarcoma of the lung, F. W. Seymour (by invitation).

Wednesday evening, November 27. Program: treatment of nephritis, Seelye W. Little.

Tuesday evening, December 10. Program: (*a*) report of case of trauma of frontal lobe; (*b*) discussion of recent advances in problem of aphasia and localisation, with lantern illustrations of serial sections, Adolph Meyer, of Pathological Institute of the State Hospitals of the State of New York (by invitation).

---

ACTIVE preparations for the International Congress on Tuberculosis to be held in Washington next September, are under way in other countries. The National Committees for France, Germany, Sweden, Austria, Holland, Greece, Bulgaria, Cuba, Venezuela, Brazil and Costa Rica have organised and have forwarded their membership lists to the secretary-general. The French committee has a membership of over three hundred and includes men of prominence in public life as well as in the medical profession. The officers of this committee are president, Dr. Louis Landouzy of the medical faculty of the University of Paris; vice presidents, Dr. Faisans of the University of Paris, Prof. Valleé, the eminent veterinarian, of Alfort, Dr. F. Bezancon of the University of Paris, and Dr. Le Gendre; secretaries, Dr. Triboulet, Secretary-General of the last International Congress, which was held in Paris three years ago, Dr. Nobecourt, Dr. Leon Bernard, Dr. Dehan, and Dr. Georges Bourgeois; treasurer, M. Masson.

The Secretary of the German Committee Dr. Johannes Nietner, was Secretary-General of the recent International Congress on Hygiene and Demography. Other members of the Committee are Dr. Gotthold Pannwitz, Secretary-General of the International Tuberculosis Association, Dr. B. Frankel, Dr. Ernst von Leyden, Professor emeritus of the University of Berlin, and Dr. Johannes Orth, Professor of Pathology in the University of Berlin.

Dr. N. P. Tenderloo, of Leyden, another well-known pathologist is a member of the Committee for Holland. Dr. P. K. Pell, of the University of Amsterdam, is chairman of that committee, and Dr. W. J. von Gorcum of The Hague is the secretary.

Dr. A. Herrera Vegas, the Chairman of the Venezuelan committee is president of the Venezuelan Anti-tuberculosis League, and a member of the National Academy of Medicine at Catacas. Dr. P. Acosta Artiz, the vice-president, is a director of the hospital at Vargas, and Dr. L. Razetti another member of the committee is vice-rector of the University of Venezuela, and permanent secretary of the National Academy of Medicine. All of the members of Brazilian committee are actively identified with the anti-tuberculosis movement in that country. The Committee includes Dr. J. J. Azevedo Lima, of Rio Janeiro, President of the Brazilian Anti-tuberculosis League; Dr. Oswaldo Cruz, Director-General of the Department of Public Health; Dr. J. J. Seabra and Dr. Cypriano de Freitas, of Rio de Janeiro.

The president of the Cuban Committee is Dr. Guiteras formerly professor of pathology in the University of Pennsylvania and now at the University of Havana. Dr. J. L. Jacobsen the vice-president is president of the Cuban Antituberculosis League. The secretary is Dr. M. G. Lebredo of Havana. Two well-known members of this committee are Dr. Aristides Agramonte, the last surviving member of the famous yellow fever commission of the United States Army, and Dr. Carlos J. Finlay who was recently awarded the Mary McKinsley medal by the Liverpool Association for the Study of Tropical Diseases.

Dr. B. Patrikios. the Chairman for the committee for Greece is Secretary of the Department of Health of Greece, and Secretary-General of the Greek Red Cross Society. Dr. Aristote Kouzis, the Secretary is a professor of the University of Athens. Dr. Constant Savas, a member of the Committee is Professor of Hygiene in the University of Athens, and is physician to the King of Greece; Dr. P. Manoussos is the principal medical director of the military hospital at Athens; Dr. Kalliontzis is professor of surgery and Dr. Pierre J. Rondopoulo is professor of pathology at the University of Athens.

The Hon. Otto von Printzkold, the Chairman of the Swedish Committee, is the first chamberlain of the Swedish court. The secretary, Dr. Bertil Buhre, is the president of the great Swedish Anti-tuberculosis League, the largest volunteer association of the kind in existence.

The Costa Rican Committee has named Dr. Louis P. Jiminez chairman, and Dr. Teodoro Picado, of San Jose, secretary. Other members are Dr. Teodoro Prestinary, Dr. Benjamin Hernandez and Dr. Marcos Zunega, all of San Jose.

Three chairmen have been named by the Austrian Committee. They are Prof. Leopold v. Schrotter, of the Medical Faculty of the University of Vienna; Dr. Weichselbaum and Dr. Richard Paltauf, of the Department of Pathology of the University of Vienna. The secretaries are Dr. H. v. Schrotter, Dr. L. Teleky and Dr. J. D. Bartel.

Dr. M. Rousseff, director of the Department of Health of Sophia, is president of the Bulgarian Committee; Dr. Ivan Oggnianoff, secretary of the Superior Board of Health at Sophia, is Secretary, and the members include Dr. Georghi Zolotovitch, Dr. Ivan Theororoff, director of the Sanatorium for Tuberculosis at Trojan, and Dr. S. A. Valcovitch.

---

The Sixteenth International Medical Congress at Budapest in 1909. The fifteenth International Medical Congress held in Lisbon, have chosen Budapest, the capital and residence of Hungary, for the site of their next assembly, and the preliminaries are already in process.

His Imperial and Apostolic Royal Majesty the King has graciously taken upon himself the patronage of the ensuing congress. The state and town have each contributed 100,000 crowns to defray the expenses.

The committee for the organisation, execution, disbursements and reception, as also for the sections is already formed and the statutes are drawn up. There are 21 sections, each branch of science having a separate section assigned to it. The date of the opening is fixed for August 29, 1909. and the sessions will be continued till September 4.

There is every reason to presume that the congress will be well attended. Hitherto they have shown an attendance of from 3,000 to 8,000 participants. Judging from the geographical situation of Budapest, at least from 4,000 to 5,000 participants may safely be reckoned upon.

The managers of course, attach the utmost importance to the scientific activity of the congress, and every effort is being made to win over the most prominent representatives of medical science.

The first circular, which will contain every necessary information as well as the statutes, will be ready for circulation in the course of the year 1907. Meanwhile the Secretary-general of the congress (Sixteenth International Medical Congress, Budapest, Hungary, VIII., Esterhazy-utcza 7), will have much pleasure in giving information to inquirers.

PROGRESS along the lines connected with the International Congress on Tuberculosis which is to take place in Washington from September 21 to October 12, 1908, was shown by the reports presented at a meting of the committee of arrangements, held in New York, at the Associated Charities Building, Monday evening, October 28. Dr. Lawrence F. Flick of Philadelphia, chairman of the committee presided, and the other members present were Dr. Joseph Walsh, Philadelphia, secretary; Dr. John S. Fulton, Washington, secretary-general; Mr. William H. Baldwin, Washington; Dr. Hermann M. Biggs, New York; Dr. Frank Billings, Chicago; Mr. Edward T. Devine, New York; Mr. Livingston Farrand, New York; Dr. J. C. Greenway, Greenwich, Conn.; Dr. Charles J. Hatfield, Philadelphia; Dr. Abraham Jacobi, New York; Dr. Alfred Meyer, Mrs. James E. Newcombe, New York; Gen. George M. Sternberg, Washington, and Dr. William H. Welch, Baltimore.

The meeting was the first held since Dr. Flick's return from abroad and his reports of his visits to the International Conference on Tuberculosis in Vienna and to the International Congress on Hygiene and Demography, at Berlin, were interesting features of the session. More than a thousand delegates were registered at Vienna, he said, and the gathering at Berlin was quite as large. The leading men in both associations are looking forward with a great deal of enthusiasm, Dr. Flick said, to the meeting in Washington, next year, and about four hundred of the members of the foreign organizations may be expected to attend the Congress. The Conference selected this country as its place of meeting in 1908 just as the Congress did two years ago. The Conference and the Congress are two distinct organizations. The International Conference on Tuberculosis meets every year and keeps up a continuous organization with headquarters in Berlin. The International Congress on Tuberculosis meets only once in three years and does not maintain an international bureau in the internals. Dr. Flick stated that at the International Conference, interest centered especially in the time-worn subject of the routes of invasion for the tubercle bacillus. It seems to have been demonstrated that the disease may be contracted by both the respiratory route, and the alimentary route. Though this does not make us much wiser in a practical way, still it is somewhat comforting to know that the respiratory route is less important than it was once thought to be. On the other hand that information is compensated by the importanceof the alimentary route.

In connection with the account of the progress made in the preliminary arrangements for the International Congress on Tuberculosis, Dr. John S. Fulton, the secretary-general, reported that ten distinguished foreigners have consented to participate in the series of special addresses that are to form a part of the program. The names of these eminent specialists follows: Dr. R. W. Philip, Edinburgh; Dr. C. Theodore Williams, London; Dr. Arthur

Newsholme, health officer, Brighton, England; Dr. C. H. Spronck, Utrecht, Holland; Dr. Karl Turban, Davos-Platz, Switzerland; Dr. Gotthold Pannwitz, Charlottenburg; Dr. Emil von Behring, Marburg; Dr. A. Calmette, Pasteur Institute, Lisle, France; Dr. Maurice Letulle, Paris; and Dr. S. Kitasato, Tokyo, Japan.

Reporting on the formation of State committees, the secretary-general said that such committees had been appointed in nearly all of the States in the United States; that several have already organised and are earnestly at work. He reported also that replies have been received from various foreign countries in reference to the appointment of committees, and the replies indicate that the countries addressed will be represented in nearly every instance by exhibits as well as by delegates.

